# Supplementary material for: Perioperative Wearable Device Features are associated with Moderate-to-Severe Chronic Pain after Surgery in the “All of Us” Research Program
Source: medRxiv. 2025 Sep 25:2025.09.23.25336489. Preprint. [Version 1] doi: 10.1101/2025.09.23.25336489 (PMC12485980; doi:10.1101/2025.09.23.25336489)
Supplement: 1 [file NIHPP2025.09.23.25336489V1-supplement-1.pdf]

medRxiv preprint doi: https://doi.org/10.1101/2025.09.23.25336489; this version posted September 25, 2025. The copyright holder for this preprint (which was not certified by peer review) is the author/funder, who has granted medRxiv a license to display the preprint in perpetuity.

It is made available under a CC-BY 4.0 International license .

Appendix

Supplemental Table 1. Overview of Fitbit-derived features, including category, feature definition, metrics, and data availability.

Category	Feature	Metric	Description	Availability
**Physical Activity**	Step count	Mean, SD, Slope	Total steps per day	All
**Physiology**	Resting heart rate	Mean, SD, Slope	Average daily heart rate	All
**Sleep**	duration	Mean, SD, Slope	Minutes asleep per day	All
	Efficiency	Mean, SD, Slope	Proportion of time asleep while in bed	All
	Fragmentation	Mean, SD, Slope	% time awake/restless/wake while in bed	All
	REM stage proportion	Mean, SD, Slope	Proportion of sleep spent in REM stage	Stages only
	deep stage proportion	Mean, SD, Slope	Proportion of sleep spent in deep stage	Stages only
	light stage proportion	Mean, SD, Slope	Proportion of sleep spent in light stage	Stages only

medRxiv preprint doi: https://doi.org/10.1101/2025.09.23.25336489; this version posted September 25, 2025. The copyright holder for this preprint (which was not certified by peer review) is the author/funder, who has granted medRxiv a license to display the preprint in perpetuity.

It is made available under a CC-BY 4.0 International license .

Supplemental Table 2. Descriptive statistics of Fitbit-derived features in the pre- and postoperative periods, stratified by pain group.

Variable	Level	No-to-low Pain (n = 215)	Moderate-to-severe Pain (n = 87)	p-value
**Physical Activity (Step Counts)**				
Mean (pre)		7903.43	6550.90	0.009
SD (pre)		3057.91	2563.02	0.004
Trend (pre)		-6.66	0.55	0.237
Days available (pre)		53.86	53.13	0.636
Mean (post)		7290.38	6210.25	0.007
SD (post)		2889.29	2455.90	0.012
Trend (post)		32.50	18.62	0.104
Days available (post)		51.47	48.32	0.089
**Resting Heart Rate Features**				
Mean (pre)		75.21	77.89	0.016
SD (pre)		3.89	4.20	0.090
Trend (pre)		-0.01	0.01	0.098
Days available (pre)		48.72	46.55	0.310
Mean (post)		75.16	78.85	0.001
SD (post)		3.92	4.66	<0.001
Trend (post)		0.01	0.04	0.525
Days available (post)		47.95	43.16	0.022
**Total Sleep Duration**				
Mean (pre)		392.24	374.71	0.016
SD (pre)		75.37	79.71	0.161
Trend (pre)		-0.31	-1.07	0.327
Days available (pre)		42.46	39.22	0.143
Mean (post)		395.56	379.44	0.029
SD (post)		73.99	85.79	<0.001
Trend (post)		-0.51	0.19	0.237
Days available (post)		41.10	34.77	0.007
**Sleep Efficiency**				
Mean (pre)		0.89	0.89	0.302
SD (pre)		0.03	0.04	0.509
Trend (pre)		< 0.001(-)	< 0.001(-)	0.855
Mean (post)		0.89	0.89	0.531

medRxiv preprint doi: https://doi.org/10.1101/2025.09.23.25336489; this version posted September 25, 2025. The copyright holder for this preprint (which was not certified by peer review) is the author/funder, who has granted medRxiv a license to display the preprint in perpetuity.

It is made available under a CC-BY 4.0 International license .

SD (post)	0.03	0.04	0.003
Trend (post)	< 0.001(+)	< 0.001(-)	0.091
**Sleep Fragmentation**			
Mean (pre)	0.12	0.13	0.691
SD (pre)	0.04	0.05	0.457
Trend (pre)	< 0.001(-)	< 0.001(+)	0.350
Mean (post)	0.13	0.12	0.546
SD (post)	0.04	0.05	0.017
Trend (post)	< 0.001(-)	< 0.001(+)	0.532
**Sleep Stages**			
REM Mean (pre)	0.20	0.19	0.056
REM SD (pre)	0.06	0.07	<0.001
REM Trend (pre)	< 0.001(-)	< 0.001(-)	0.321
REM Mean (post)	0.20	0.20	0.446
REM SD (post)	0.06	0.07	0.017
REM Trend (post)	< 0.001(-)	< 0.001(-)	0.094
Light Mean (pre)	0.65	0.65	0.643
Light SD (pre)	0.08	0.09	<0.001
Light Trend (pre)	< 0.001(-)	< 0.001(+)	0.112
Light Mean (post)	0.65	0.65	0.535
Light SD (post)	0.08	0.09	0.175
Light Trend (post)	< 0.001(-)	< 0.001(+)	0.068
Deep Mean (pre)	0.15	0.15	0.080
Deep SD (pre)	0.05	0.05	0.114
Deep Trend (pre)	< 0.001(+)	< 0.001(-)	0.158
Deep Mean (post)	0.14	0.15	0.027
Deep SD (post)	0.05	0.05	0.061
Deep Trend (post)	< 0.001(+)	< 0.001(-)	0.581

Sleep stage features are based on participants with stage-mode devices (n = 244). Continuous variables are summarized as mean (SD = Standard Deviation), and “trend” reflects the linear slope over time. For sleep efficiency and fragmentation, slopes extremely close to zero are reported as “<0.001,” with a positive (+) or negative (-) sign to indicate the direction of change. Continuous variables were compared pain groups using Welch’s two-sample t-tests. P-values are reported to three significant digits; continuous summaries are shown to two decimals.

medRxiv preprint doi: https://doi.org/10.1101/2025.09.23.25336489; this version posted September 25, 2025. The copyright holder for this preprint (which was not certified by peer review) is the author/funder, who has granted medRxiv a license to display the preprint in perpetuity.
It is made available under a CC-BY 4.0 International license .

Supplemental Table 3. Comparison of model performance across demographic and multivariate models.

Model	Train AUC	Test AUC	AIC	Sensitivity	Specificity
Demographics (Base model)	0.636	0.584	243.53	0.032	1.00
Physical Activity (Step counts)	0.695	0.677	248.73	0.10	0.95
Heart Rate	0.724	0.596	239.65	0.23	0.90
Sleep Features	0.798	0.722	241.15	0.42	0.92
Sleep Stage Features	0.799	0.652	207.59	0.19	0.87
Multivariate (All)	0.753	0.679	239.92	0.26	0.92
Multivariate (All - stepwise)	0.720	0.648	227.07	0.26	0.92
Multivariate (Stage-only)	0.816	0.671	201.83	0.44	0.87
Multivariate (Stage-only - stepwise)	0.781	0.649	178.75	0.37	0.87

Multivariate (All) included the full cohort (n = 302), while Multivariate (Stage-only) only included participants with sleep stage-mode data available (n = 244).
